# Complement-Opsonized HIV-1 Alters Cross Talk Between Dendritic Cells and Natural Killer (NK) Cells to Inhibit NK Killing and to Upregulate PD-1, CXCR3, and CCR4 on T Cells

**DOI:** 10.3389/fimmu.2018.00899

**Published:** 2018-04-30

**Authors:** Rada Ellegård, Mohammad Khalid, Cecilia Svanberg, Hanna Holgersson, Ylva Thorén, Mirja Karolina Wittgren, Jorma Hinkula, Sofia Nyström, Esaki M. Shankar, Marie Larsson

**Affiliations:** ^1^Division of Molecular Virology, Department of Clinical and Experimental Medicine, Linköping University, Linköping, Sweden; ^2^Department of Pharmaceutics, College of Pharmacy, King Khalid University, Asir-Abha, Saudi Arabia; ^3^Clinical Immunology and Transfusion Medicine, Department of Clinical and Experimental Medicine, Linköping University, Linköping, Sweden; ^4^Division of Infection Biology, Department of Life Sciences, Central University of Tamil Nadu, Thiruvarur, India; ^5^Center of Excellence for Research in AIDS (CERiA), University of Malaya, Lembah Pantai, Kuala Lumpur, Malaysia; ^6^Department of Microbiology, Central University of Tamil Nadu, Thiruvarur, India

**Keywords:** dendritic cells, natural killer cells, complement, HIV, cross talk, checkpoint inhibitors, CXCR3, CCR4

## Abstract

Dendritic cells (DCs), natural killer (NK) cells, and T cells play critical roles during primary HIV-1 exposure at the mucosa, where the viral particles become coated with complement fragments and mucosa-associated antibodies. The microenvironment together with subsequent interactions between these cells and HIV at the mucosal site of infection will determine the quality of immune response that ensues adaptive activation. Here, we investigated how complement and immunoglobulin opsonization influences the responses triggered in DCs and NK cells, how this affects their cross talk, and what T cell phenotypes are induced to expand following the interaction. Our results showed that DCs exposed to complement-opsonized HIV (C-HIV) were less mature and had a poor ability to trigger IFN-driven NK cell activation. In addition, when the DCs were exposed to C-HIV, the cytotolytic potentials of both NK cells and CD8 T cells were markedly suppressed. The expression of PD-1 as well as co-expression of negative immune checkpoints TIM-3 and LAG-3 on PD-1 positive cells were increased on both CD4 as well as CD8 T cells upon interaction with and priming by NK–DC cross talk cultures exposed to C-HIV. In addition, stimulation by NK–DC cross talk cultures exposed to C-HIV led to the upregulation of CD38, CXCR3, and CCR4 on T cells. Together, the immune modulation induced during the presence of complement on viral surfaces is likely to favor HIV establishment, dissemination, and viral pathogenesis.

## Introduction

The microenvironment and the interactions between immune cells and pathogens at the site of infection determine the quality of the immune responses activated. At the mucosal site of primary HIV infection, dendritic cells (DCs) represent one of the first cell types that interact with the virus particles (1, 2). DCs are part of both the innate and the adaptive immune responses and have the ability to sense danger, such as presence of HIV, and send out signals that alert the body to fight infection and also to induce specific T cell-mediated adaptive immune responses directed against the virus (3). However, in addition to playing an essential part in host defense, DCs also play a dexterous role by enhancing viral spread to newly activated CD4 T cells in the submucosa and lymph nodes, which is a key step in the establishment of systemic HIV infection (4). The initial events and responses generated during acute retroviral exposure across the mucosal surfaces are key to establishment of viral set point and rate of HIV disease progression, and DCs are important determinants of these responses (5).

Body fluids, such as semen, vaginal secretions, and breast milk, in contact with HIV particles will cover the virus with soluble factors, including complement components and antibodies. Complement-mediated lysis of HIV is largely inefficient due to presence of regulators of complement activation across the viral membrane, resulting in virus opsonization by inactivated complement fragments, such as iC3b (6), which protect HIV from lysis and enhance the infectivity when it comes to both direct infection of DCs and transfection from DCs to T cells (7–9). The inactivated complement fragment iC3b has been shown to interact with complement receptor 3 (CR3) expressed on DCs, resulting in the promotion of HIV phagocytosis (10) and modulation of antigen presentation (11). We demonstrated that enhancement of infection in DCs is largely due to complement-mediated suppression of inflammation and antiviral responses *via* CR3 (12). Furthermore, we also found that the ability of DCs to attract other innate immune cells, especially natural killer (NK) cells to the site of infection was impaired when the DCs were exposed to complement-opsonized HIV (C-HIV) as a result of suppressed production of chemoattractants, including CCL3 and CXCL10 (13). In addition, it has also been suggested that CR3 engagement of DCs decreases their capacity to stimulate T cells (14).

The importance of functional NK cell responses is exemplified by the ability of these cells to control SIV replication in the lymph nodes they relocate to in SIV-infected animals (15). The diminished recruitment of NK cells to the site of infection appears to accentuate the establishment of HIV infection, seeing that NK cells have been shown to directly restrict viral spread by killing infected cells and indirectly by secreting antiviral factors (16, 17). In addition, NK cells can also produce inflammatory cytokines, such as IFN-γ, which promote further activation of innate and adaptive immune responses (18). The ability of NK cells to kill infected cells is key to impediment of HIV-infection (16, 17). The necessity of NK cells for HIV protection and control is further illustrated by the fact that loss of NK cell functions is associated with poor disease prognosis (19) and the correlation between protection against infection and the level of NK cell activity in HIV-exposed uninfected individuals (20). In HIV-infected individuals, there is a dysfunctional population of NK cells with reduced cytokine production and cytolytic activity (21, 22). NK cell dysfunction also appears to influence the immune activation potential of DCs, which affects the ensuing T-cell responses (22).

In order to necessitate protective antiviral immune responses, DCs must receive optimal activation and maturation signals. The cross talk between DCs and NK cells can have either positive or negative effects on the respective cell’s functionality. When interaction between these cells occurs in a setting where there is a high proportion of NK cells per DC, this can result in high level of lysis of DCs (16, 17). In settings with a low NK cell to DC ratio, the NK–DC cell interactions enhance the expression of activation markers, e.g., MHC class II, CD80, CD86, and increase the synthesis of IL-12 by DCs (23). Thus, support from NK cells is imperative for proper DC maturation (24, 25) and the DC maturation depends on cell–cell interaction between DCs and NK cells, and may possibly involve the association of NKp30 receptor and production of TNF and IFN-γ by NK cells (26). While the DCs contribute by releasing IL-18, which triggers HMGB1 secretion by NK cells that further enhances the DC maturation process.

The NK–DC cross talk will also influence subsequent development of T cell responses, with the NK cell IFN-γ production affecting both the CD4 and CD8 T cell responses (27). HIV susceptibility is influenced both by the availability as well as the phenotypes of target cells present across the mucosa (28). Hence, T cell migration as well as phenotypes that are induced as a result of initial NK cell and DC responses during HIV transmission is likely to have an important impact on the outcome of infection.

There are numerous studies on the direct effects of free HIV on single cultures of DCs, NK cells, and T cells, and here, we aimed to investigate how the virus, and presence of complement on its surface, affects the interactions between them. The effects C-HIV exerts on NK cells directly and on the NK–DC cross talk have to our knowledge never been investigated previously. We found that complement opsonization of HIV altered DC responses in a way that suppressed NK activation and their killing ability. In addition, NK–DC cross talk in the presence of C-HIV generated T cells with a higher expression of immune checkpoint inhibitors such as PD-1, and chemokine receptors CXCR3 and CCR4. The observed immune modulation is likely to aid HIV in establishing infection in the host and contributes to HIV pathogenesis.

## Materials and Methods

### Virus Generation and Opsonization

HIV-1BaL (lot no. 4238) was produced from SUP-T 1/CCR5 cells and purified as described previously (29). The virus (30 ng/µl) was incubated for 1 h with either an equal volume of RPMI 1640 (Sigma-Aldrich, Stockholm, Sweden) to generate free HIV (F-HIV), single-donor human serum, to generate C-HIV, or single-donor human serum supplemented with 2 µg/ml HIV-specific IgG and 20 µg/ml γ-globulins, to generate complement- and antibody-opsonized HIV (CI-HIV).

### Cell Purification and Culture

Monocyte-derived DCs were prepared and cultured as described previously (30). In brief, PBMCs were separated from whole blood from healthy volunteers (Ethical approval No. EPN 173-07), *N* = 30. DC progenitors were enriched from the PBMCs by plastic adhesion to tissue culture plates for 1 h at 37°C. The non-adherent cells were harvested and cryopreserved for subsequent purification of NK cells, or were used directly for purification of T cells. Adherent cells were cultured in RPMI1640 with l-glutamine supplemented with 10 mM HEPES, 20 µg/ml gentamicin (Fisher Scientific, Leicestershire, UK), 100 IU/ml recombinant human GM-CSF, 300 U/ml recombinant human IL-4 (Prepotech, UK), and 1% human plasma for 5 days. The DC purity and maturation status was assessed by flow cytometry staining for CD14 and CD83. All DC preparations used were more than 98% pure and had less than 5% CD14 and CD83 expression. Pan-T cells were purified from fresh non-adherent cells by negative magnetic bead purification using a commercial human Pan-T cell Isolation kit (Miltenyi Biotec, Lund, Sweden) according to the manufacturer’s protocols. Memory T cells were depleted using CD45RO microbeads (Miltenyi Biotec). NK cells from frozen non-adherent cells were purified the same day as they were added to the NK–DC cross talk assays using a commercial human NK cell Isolation kit (Miltenyi Biotec) according to the manufacturer’s instructions. NK cells and DCs from the NK–DC cross talk assays were separated by positive selection of CD1c+ DCs using microbeads separation (Miltenyi Biotec).

### NK–DC Cross Talk Assay

**Figure 1:**
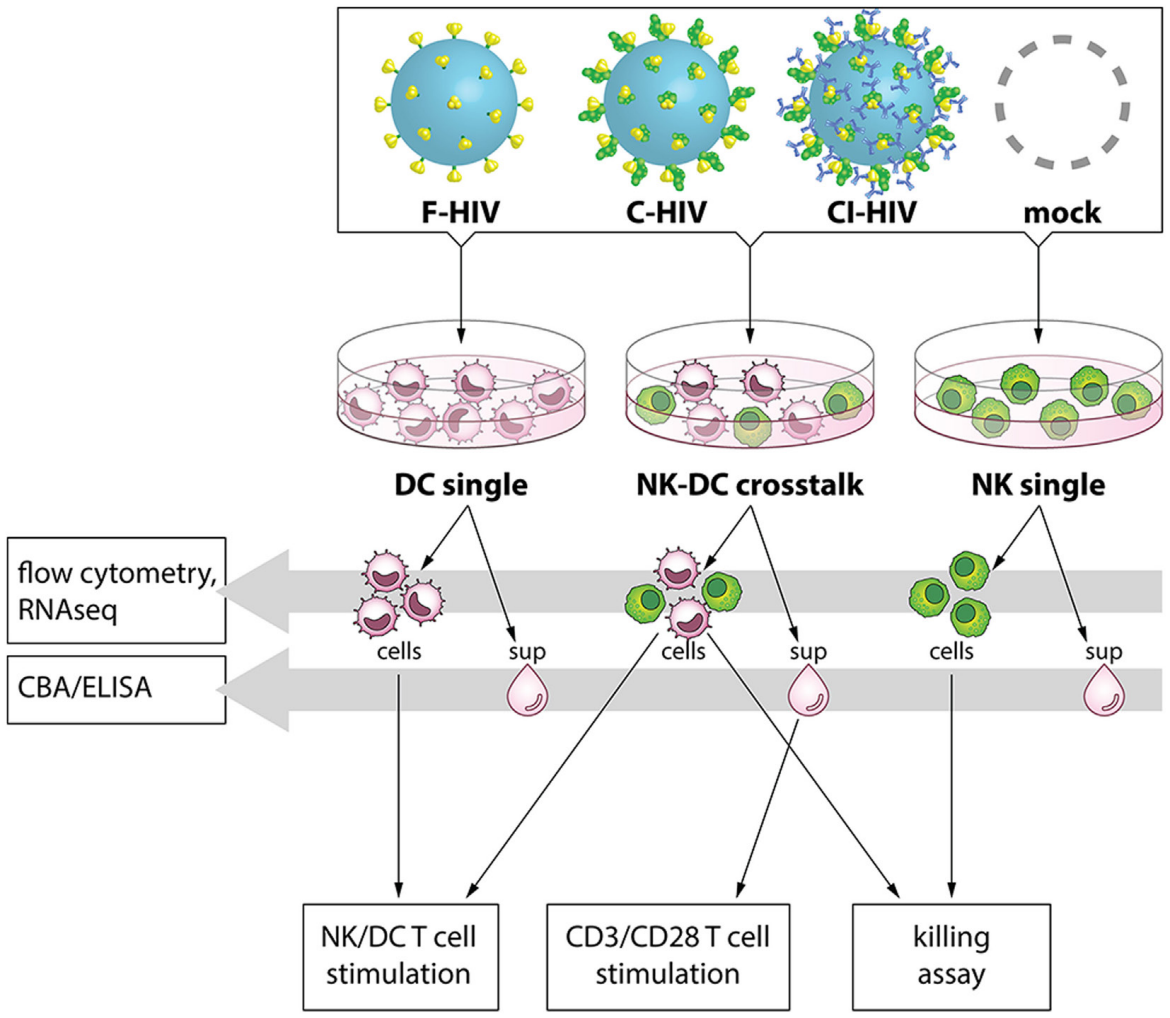
Experimental study design. Dendritic cells (DCs) and natural killer (NK) cells were kept in single cultures, or in cross talk cultures consisting of NK cells and DCs from the same donor at a 1:1 ratio and exposed to 1 μg/ml F-HIV, complement-opsonized HIV (C-HIV), complement- and antibody opsonized HIV (CI-HIV), or mock treated for 24 h. Flow cytometry was performed to assess expression of activation markers on the cells, and the pathways activated were assessed by RNAseq. The concentration of cytokines in the supernatants was measured using a cytometric bead array (CBA) or ELISA. Allogeneic T cells were stimulated using cells from the cultures, or by CD3/CD28 ligation together with culture supernatants. The proliferation and the phenotype of the T cells was subsequently evaluated.

Autologous DCs and NK cells from the same donor were exposed to mock, i.e., exposed to the same media as added to the HIV preparation, 100 ng/1 × 10^5^ cells F-HIV, C-HIV, or CI-HIV for 24 h, either in single cultures or in NK–DC cross talk cultures. For the NK–DC cross talk cultures, NK cells were added to DC cultures 3 h post HIV exposure at a 1:1 ratio followed by additional 21 h incubation. An overview of the cross talk assays and the design of this study can be found in Figure 1.

### Cytometric Bead Array (CBA) and ELISA

The protein levels of IFN-γ, IL-6, IL-12, CXCL10, IL-5, CX3CL1, CXCL9, CCL3, and CCL4 in cell supernatants were assessed using a commercial CBA (BD Biosciences, Stockholm, Sweden) performed on a BD FACSCanto II flow cytometer (BD Biosciences) and analyzed using a FCAP Array version 3 software (BD Biosciences) according to the manufacturer’s protocols. Furthermore, the protein levels of IL-15 (Sino Biological, NordicBiosite, Stockholm, Sweden) and IL-23 (Mabtech, Stockholm, Sweden) were measured using commercial ELISA kits according to the manufacturer’s protocols.

### RNA Sequencing and Data Handling

Whole transcriptome amplification of RNA purified from DCs and NK cells from single and NK–DC cross talk cultures was done using NuGEN’s Ovation RNA-Seq V2 kit following the protocol provided by the company (San Carlos, CA, USA). cDNA was amplified from total RNA using a single primer isothermal amplification and purified using a MinElute Reaction Cleanup Kit (Qiagen; Valencia, CA, USA). The cDNA samples were fragmented, barcoded with adaptors, and amplified using an Ultralow System V2 kit. Distribution of the size of the library was determined using an Agilent Bioanalyzer 2100. Libraries from three different donors were sequenced on the Illumina NextSeq500 platform (San Diego, CA, USA). The fastq files were uploaded and the quality checked using fastQC (31). Trimmomatic (32) was used to remove adaptors and low-quality bases and the reads were then mapped to human reference genome hg19 using STAR. FeatureCounts was used to calculate counts for each gene (33). The data were normalized and R/DeSeq2 used to determine differentially expressed genes (34). Analysis of pathways was done by Ingenuity Pathway Analysis (Qiagen), R analysis, and custom gene lists.

### NK Cell Killing Assay

K562 cells (ATCC, Manassas, VA, USA) were cultured in RPMI 1640 supplemented with 10% FBS, 20 µg/ml gentamicin, and 10 mM HEPES (Fisher Scientific, Leicestershire, UK) and used as target cells for killing by NK cells from single or purified from NK–DC cross talk cultures exposed to mock, F-HIV, C-HIV, or CI-HIV. PHA (1 μg/ml)-activated NK cells were used as positive controls. The frequency of CFSE-labeled K562 cells undergoing cell death following incubation with NK cells at a 1:8 ratio for 6 h was determined using a BD FACSCanto™ II flow cytometer and analyzed using FlowJo™ Software (Treestar, OH, USA).

### T Cell Activation and Proliferation Assay

Allogeneic pan-T cells were added to the NK–DC cross talk cultures or to DC single cultures 24 h after stimulation with mock, F-HIV, C-HIV, or CI-HIV and cultured at a DC:T cell ratio of 1:10 in 96-well plates in 5% PHS supplemented with 10 μM azidothymidine (AZT). In addition, pan-T cells were activated with CD3/CD28 T Cell Activator according to the manufacturer’s protocol (ImmunoCult™, STEMCELL technology) in the presence of supernatant harvested at 24 h from the NK–DC cross talk or DC single cultures and cultured in 96-well plates in 5% PHS supplemented with 10 μM AZT. After 24 h exposure, the T cells were washed and re-cultured with IL-2 and 10 μM AZT. PHA stimulation (1 μg/ml) of the Pan-T cells was used as a control for T-cell activation. T-cell phenotypes were assessed by flow cytometry on day 3 (FACSCalibur, BD Immunocytometry Systems, San Jose, CA, USA). The antibodies used were AmCyan mouse anti-human CD3 (Clone SK7), FITC anti-human CD45RA (Clone HI100), PE anti-human TIM-3 (CD366) (Clone 7D3) (all from BD Biosciences). PerCP/Cy5.5 anti-human CD8, APC anti-human CD4 Brilliant Violet 421 anti-human CD4, Pacific Blue anti-human CD197 (CCR7), Alexa Fluor 647 anti-human Granzyme B, PE anti-mouse CD183 (CXCR3), Pacific Blue anti-human perforin, Alexa Fluor 647 anti-human CD194 (CCR4), PE/Cy7 anti-human CD38, FITC anti-human CD223 (LAG-3), Zombie NIR Fixable Viability Kit, PE anti-human CD366 (TIM-3), Brilliant Violet 421 anti-human CD279 (PD-1) (all from NordicBiosite), and PE-eFluor 610 anti-human CD279 (PD-1) from eBioscience. T-cell proliferation was assessed by adding 4μCi of ^3^H-Thymidine (Amersham Pharmacia Biotech) to the assay on day 4 and measuring the incorporation after 20 h.

### Phenotypic Analysis of NK Cells and DCs

Phenotypic analysis of NK cells and DCs was performed by flow cytometry. The cells were collected and resuspended in RPMI1640 supplemented with EDTA (Fisher Scientific) in order to disrupt any cell aggregates and were subsequently stained with antibodies specific for CD1c, CD25, CD40, CD56, CD69, CD80, CD86, and HLADR and their corresponding isotype controls (BD Biosciences, Stockholm, Sweden). The cells were analyzed with a FACS flow cytometer (BD Immunocytometry Systems, San Jose, CA, USA) and FlowJo software (TreeStar, AsMHCnd, OR, USA).

### Statistics

The RNA data were normalized and R/DeSeq2 used to determine differentially expressed genes. Analysis of pathways was done by Ingenuity Pathway Analysis (Qiagen), and R analysis, and custom gene lists. All other results were analyzed using Graph Pad Prism 5 (GraphPad Software, La Jolla, CA, USA), with repeated measures ANOVA followed by Bonferroni posttest. *p* < 0.05 was considered statistically significant. In all figures, *N* denotes the number of times an experiment was repeated, each time with cells derived from a different donor.

### Data Availability

The RNAseq datasets generated for this study can be found at Sequence Read Archive, accession SRP131436. The raw data supporting the conclusions of this manuscript will be made available by the authors, without undue reservation, to any qualified researcher.

## Results

### Complement Opsonization of HIV Reduced DC-Induced NK Activation During NK–DC Cross Talk

**Figure 2:**
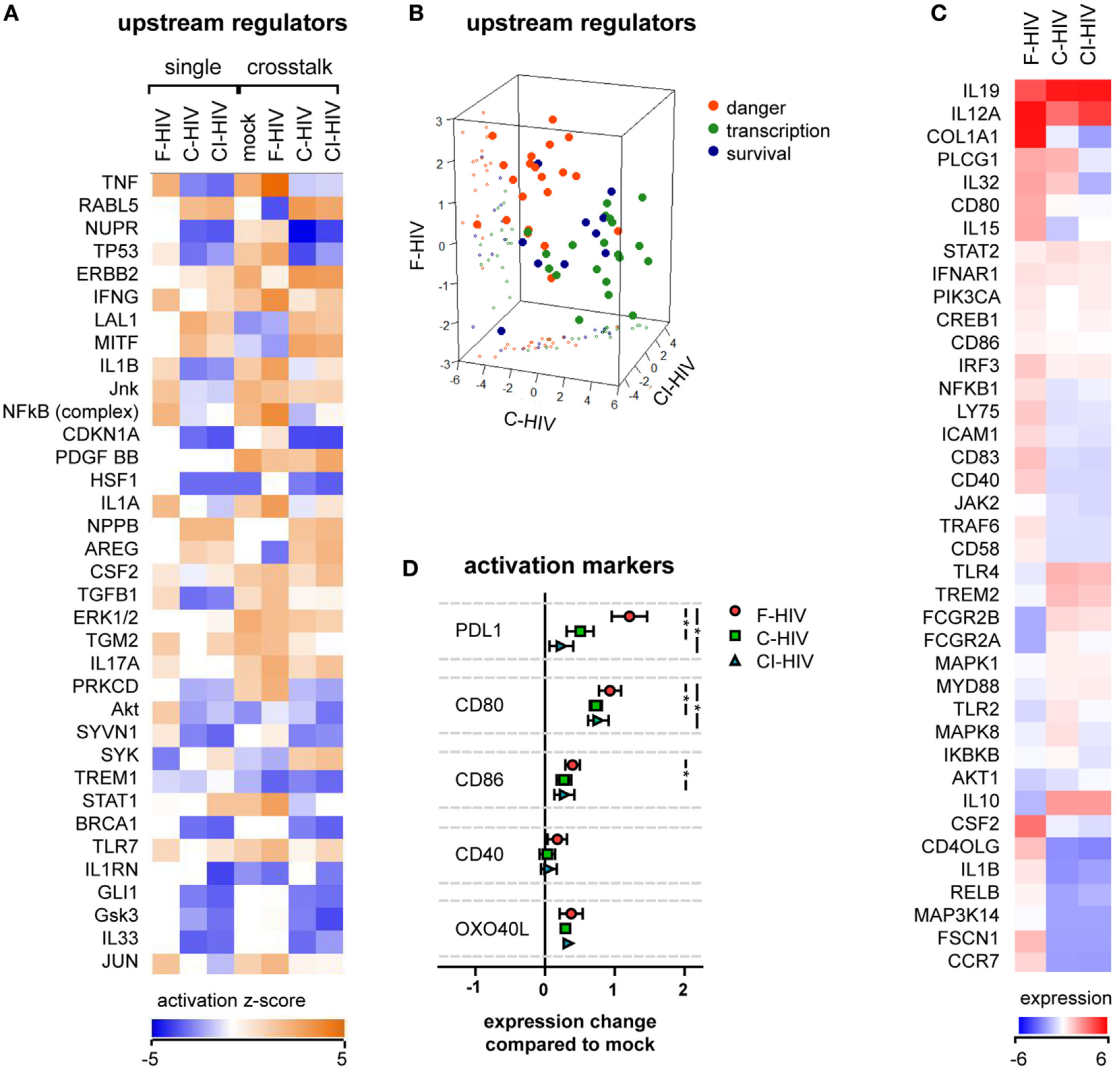
HIV effects on dendritic cell (DC) activation in the absence or presence of natural killer (NK) cells. DCs were exposed to 1 μg/ml F-HIV, complement-opsonized HIV complement- and antibody opsonized HIV (CI-HIV), CI-HIV, or mock treated either in DC single culture, or in a cross talk culture consisting of NK cells and DCs from the same donor at a 1:1 ratio for 24 h. The experiment was replicated three times using cells derived from three different donors. **(A)** RNAseq was performed on the DCs from the single culture or DCs isolated from the NK–DC cross talk, and the genes that were significantly differentially expressed were analyzed using IPA (Qiagen) to determine the most affected upstream regulators. **(B)** The top upstream regulators were divided into functional groups, and a 3D scatterplot was used to visualize clustering. **(C)** A heatmap was generated for genes that were significantly differentially expressed in at least one condition and that were defined by IPA to be involved in DC maturation. The heatmap shows fold change in the virus exposed cross talk samples compared to the mock treated cross talk samples. **(D)** The protein expression of surface activation markers on the DCs from the cross talk cultures was assessed using flow cytometry, and the values normalized to the expression in the mock-treated cross talk culture. One-way ANOVA followed by a Bonferroni posttest was used to test for statistically significant differences in protein expression. Boxplots show mean ± SEM (*p < 0.05).

Dendritic cells were preexposed to mock, F-HIV, C-HIV, or CI-HIV for 3 h. After this, DCs were maintained either as a single culture, or NK cells were added to facilitate cross talk for 21 h. RNAseq was performed on the DCs isolated from the cultures, to examine the effect of different virus exposures on the DCs and their cross talk with NK cells. Analysis of the RNAseq data using IPA revealed that in DCs, complement opsonization of HIV decreased the activation of upstream regulators associated with inflammatory and antiviral responses, such as TNF, IL-1β, NFκβ, and IFNγ, while several regulators of growth such as RABL5 and ERBB2 were increased (Figure 2A). This profile was similar for DCs derived from both single and cross talk cultures, i.e., the presence of NK cells did not have a dramatic effect on DC activation (Figure 2A). The majority of regulators fell into three main functional groups–danger (factors shown to be involved in inflammatory, antiviral, or stress responses), transcription (factors associated with GO annotation terms “transcription factor activity,” “sequence specific DNA binding,” and “chromatin binding”), and survival (factors regulating cell growth, cycle, and survival). The upstream regulators involved in danger signaling pathways showed a high positive clustering for F-HIV, whereas the upstream regulators for cell survival had a higher positive clustering for C-HIV and CI-HIV groups (Figure 2B). The list of the top regulators generated by IPA and to which group they were assigned can be found in Table S1 in Supplementary Material. The RNAseq results were filtered for genes reported to be involved in DC maturation that were significantly affected (*p* < 0.05) for at least one culture condition and normalized to the mock-treated cross talk sample (Figure 2C). Several factors involved in the maturation of DCs, such as CD40LG, IL1B, RELB, and CCR7, were upregulated in F-HIV-exposed cells, while expression of these factors in cells exposed to C-HIV or CI-HIV were downregulated (Figure 2C). When assessing the expression on protein level, the presence of HIV during the cross talk between DCs and NK cell increased the expression levels of factors involved in the DC maturation (Figure 2D). The highest impact was seen for the expression of PD-L1, a receptor for PD-1 involved in immune suppression, where F-HIV induced a significantly higher upregulation than C-HIV or CI-HIV (Figure 2D). In addition, the costimulatory molecule CD80 was upregulated on DCs exposed to F-HIV (Figure 2D).

### Activation of NK Cells Was Altered by Cross talk With HIV-Exposed DCs

**Figure 3:**
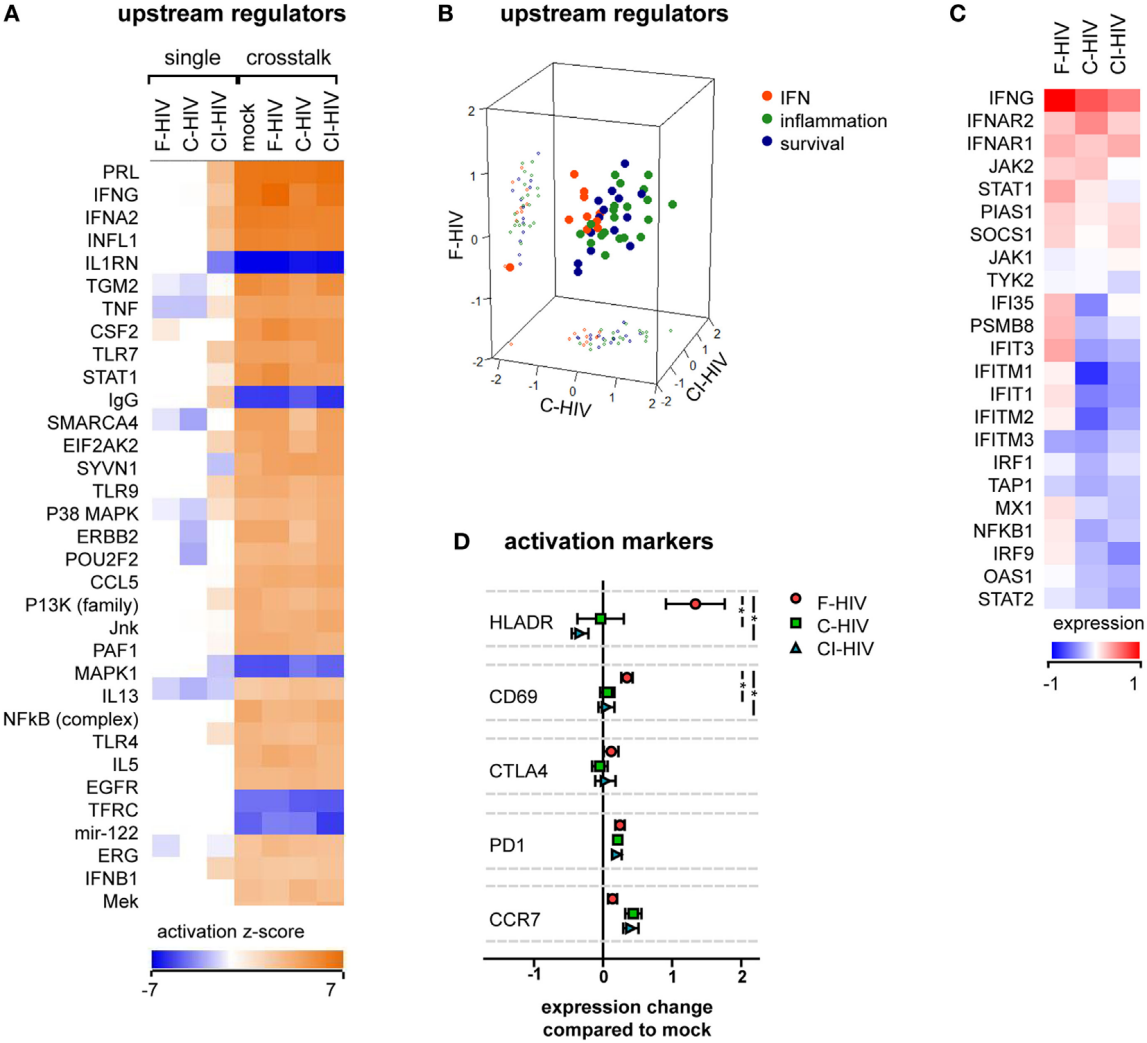
HIV effects on natural killer (NK) cell activation in the absence or presence of dendritic cells (DCs). NK cells were exposed to 1 μg/ml F-HIV, complement-opsonized HIV (C-HIV), CI-HIV either in single NK cell culture, or in a cross talk culture consisting of NK cells and DCs from the same donor at a 1:1 ratio. The experiment was replicated three times using cells derived from three different donors. **(A)** RNAseq was performed on the NK cells from the single culture or NK cells isolated from the NK–DC cross talk, and the genes that were significantly differentially expressed were analyzed using IPA (Qiagen) to determine the most affected upstream regulators. **(B)** The top upstream regulators were divided into functional groups, and a 3D scatterplot was used to visualize clustering. **(C)** A heatmap was generated for genes that were significantly differentially expressed in at least one condition and that were defined by IPA to be involved in IFN signaling. The heatmap shows fold change in the virus exposed cross talk samples compared to the mock-treated cross talk samples. **(D)** The protein expression of activation markers on the NK cells from the cross talk cultures was assessed using flow cytometry, and normalized to the expression in the mock-treated cross talk culture. One-way ANOVA followed by a Bonferroni posttest was used to test for statistically significant differences in protein expression. Boxplots show mean ± SEM (*p < 0.05).

The transcriptome profiles of NK cells from the cross talk cultures exposed to mock, F-HIV, C-HIV, or CI-HIV showed high effects on many factors involved in NK cell activation compared to the NK cell groups cultured without DCs (Figure 3A). The cross talk between the NK cell and DCs in itself had a stronger influence on NK cell activation than the HIV exposure. The top upstream regulators in the cross talk cultures according to IPA analysis fell under three main categories: IFN (factors induced by IFN or involved in IFN signaling), inflammation, and survival. The upstream regulators involved in inflammation and survival responses were upregulated in all HIV-exposed cross talk groups compared to mock, whereas regulators in the IFN group tended to be upregulated in cultures exposed to F-HIV only (Figure 3B). The list of the top regulators generated by IPA and the groups to which they were assigned in can be found in Table S2 in Supplementary Material. The RNAseq results were filtered for genes reported by IPA to be involved in IFN signaling and normalized to the mock treated cross talk NK cell sample (Figure 3C). Several factors involved in IFN signaling were higher in NK cells from cross talk cultures exposed to F-HIV compared to cultures exposed to C-HIV or CI-HIV (Figure 3C). When assessing the expression of NK cell activation markers on protein level, HLADR and CD69 were upregulated on the F-HIV-treated NK cells from the NK–DC cross talk compared to cells exposed to complement-opsonized virus. The homing receptor CCR7 was slightly more upregulated (not statistically significant) on NK cells derived cultures exposed to C-HIV and CI-HIV compared to F-HIV (Figure 3D). Next, we assessed the expression of PD-1 on NK cells in the NK–DC cocultures as this negative immune checkpoint molecule is expressed not only on T cells but also on NK cells (35–37). The expression of PD-1 on NK cells activated by the DCs in the cross talk assay was induced in the presence of any of the HIV groups (Figure 3D).

### The Cellular Cross Talk Altered the Profiles of Cytokine and Chemokine Production in Both DCs and NK

**Figure 4:**
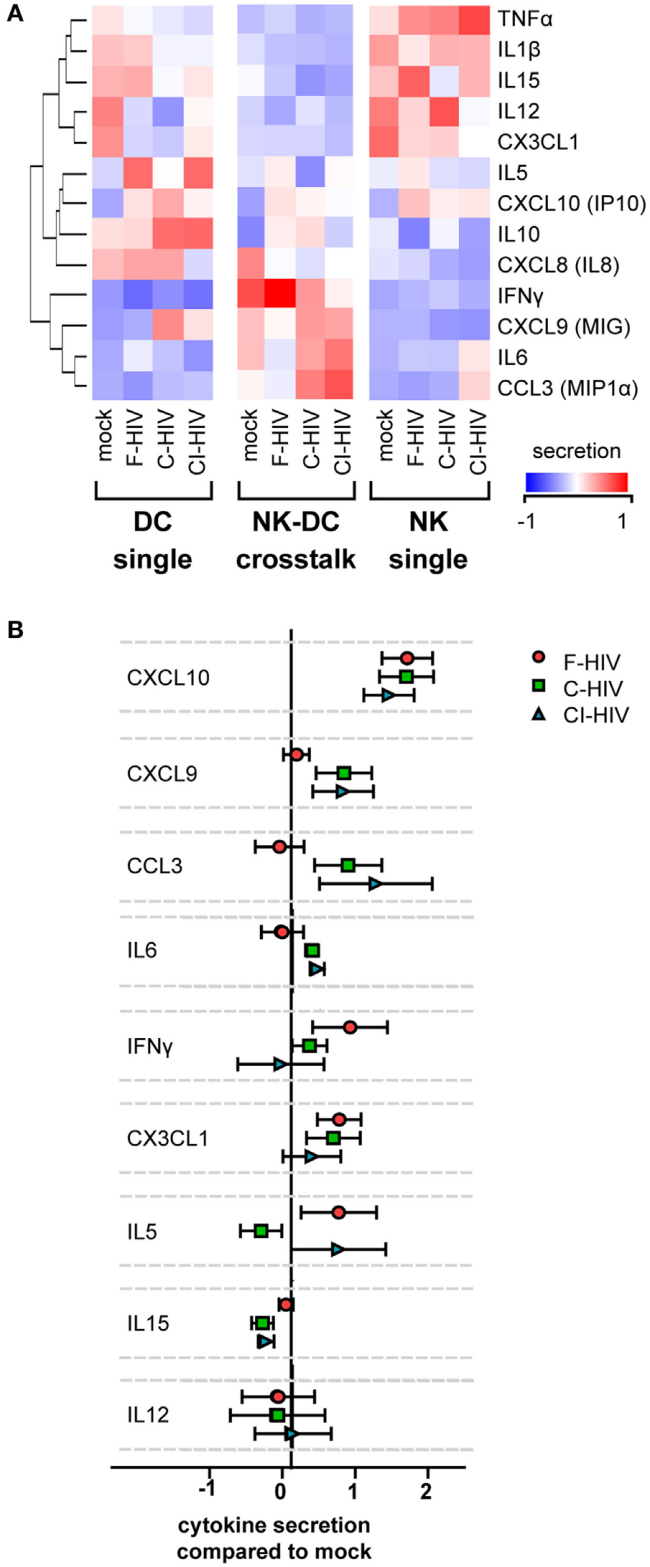
Cytokine secretion profiles in the natural killer (NK)–dendritic cell (DC) cross talk assay. DCs and NK cells were kept in single cultures, or in cross talk cultures consisting of NK cells and DCs from the same donor at a 1:1 ratio and exposed to 1 μg/ml F-HIV, complement-opsonized HIV (C-HIV), CI-HIV, or mock treated for 24 h. The experiment was replicated four times using cells derived from four different donors. **(A)** The supernatants were harvested, and the concentration of cytokines was determined using a cytometric bead array or ELISA. The values were normalized (centered) and a heat map was created. **(B)** Cytokine concentration in the NK–DC cross talk cultures were normalized to the mock treated samples. Boxplots show mean ± SEM.

Release of inflammatory and chemotactic factors into the supernatants of the single DC and NK cultures as well as from cross talk cultures was assessed using a CBA or ELISA. TNF, IL-1β, IL-15, IL-12, and CX3CL1 were produced by NK cells in single culture, but the production was downregulated during cross talk with DCs (Figure 4A). IL-5, CXCL10, IL-10, and CXCL8 were produced by DCs in single culture, and were upregulated by HIV exposure (Figure 4A). These cytokines were to some extent downregulated during cross talk with NK cells (Figure 4A). The release of IL-1β and IL-15 was downregulated by C-HIV compared to free HIV (Figure 4A). Production of IFN-γ, CXCL9, IL-6, and CCL3 were highest during cross talk compared to single cultures of both NK and DCs (Figure 4A). Secretion of the chemotactic cytokines CXCL9 (MIG) and CCL3 were higher in the NK–DC cross talk cultures exposed to C-HIV or CI-HIV compared to F-HIV (Figures 4A,B). In contrast, cytokines that have been shown to activate NK cells, such as IFN-γ, CX3CL1 (Fraktalkine), and IL-15, were higher in the cross talk cultures exposed to F-HIV than in the cultures exposed to C-HIV or CI-HIV (Figures 4A,B). IL-6 secretion was highest in the cross talk cultures exposed to C-HIV or CI-HIV (Figures 4A,B). In the cross talk cultures, IFN-γ production was higher in F-HIV and lower in C-HIV and CI-HIV compared to mock (Figures 4A,B).

### HIV Decreased the Cytotoxicity in NK Cells Activated by Cross Talk Between NK Cells and DCs

**Figure 5:**
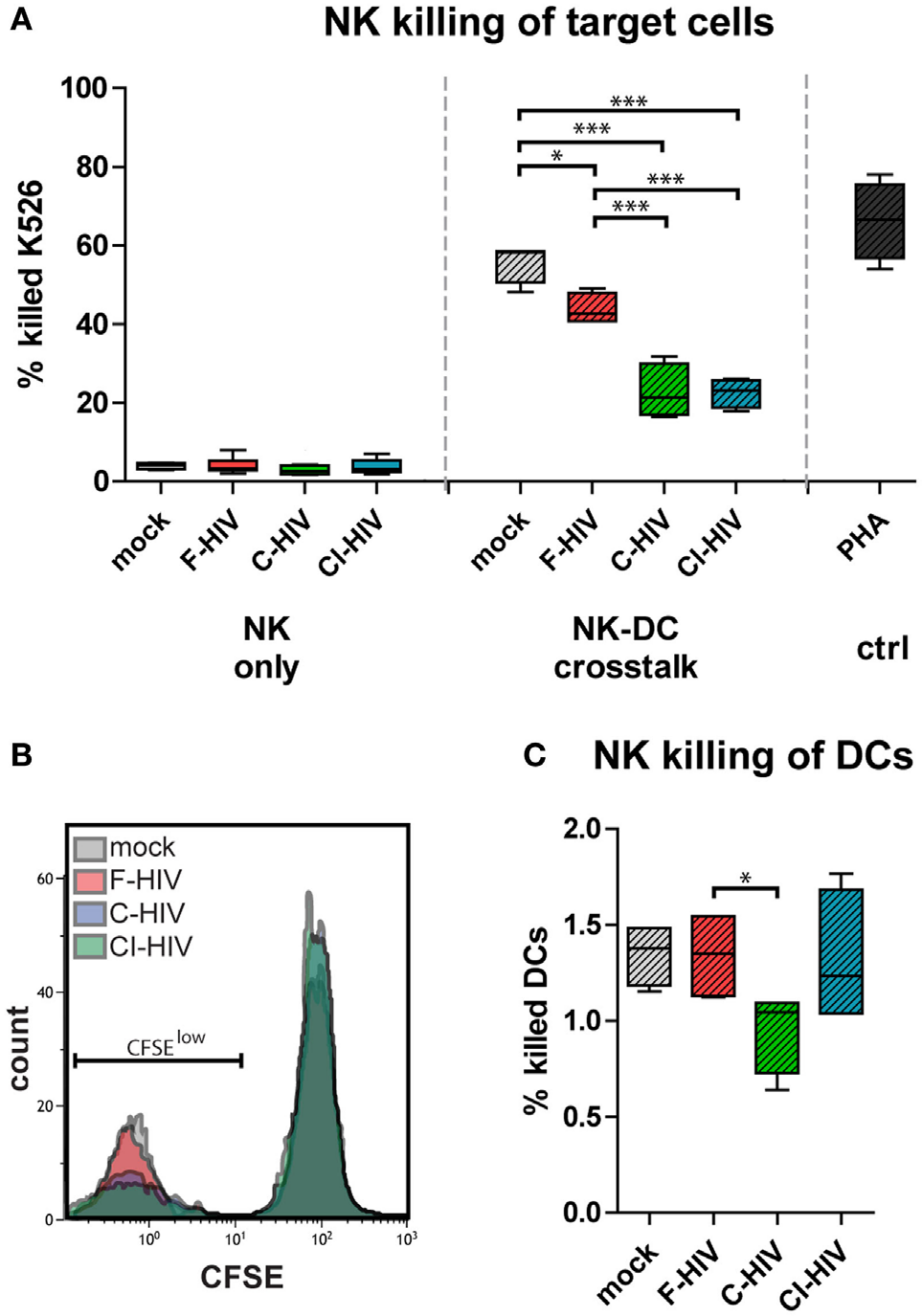
HIV decreased the cytotoxicity in natural killer (NK) cells activated by cross talk between NK cells and dendritic cells (DCs). NK cells were maintained in single NK cell cultures, or in cross talk cultures consisting of NK cells and DCs from the same donor at a 1:1 ratio and exposed to 1 μg/ml F-HIV, complement-opsonized HIV (C-HIV), CI-HIV, PHA, or mock treated for 24 h. The experiment was replicated four times using cells derived from four different donors. **(A)** NK cells from single or purified from the cross talk assays were added to target cells (CFSE-stained K562 cells) at a 1:8 ratio for 6 h and the amount of killed cells were assessed using flow cytometry. **(B)** Representative histograms for the different experimental conditions. **(C)** A similar assay was performed, using CFSE-stained DCs as target cells. Boxplots show mean with Tukey error bars. One-way ANOVA followed by a Bonferroni posttest was used to test for statistical significance (*p < 0.05, *N* = 4).

We assessed the functionality of NK cells exposed to F-HIV, C-HIV, CI-HIV, or mock either from single cultures or from NK–DC cross talk cultures. The NK cell’s ability to kill target cells (CFSE-stained K562) was assessed using flow cytometry (Figures 5A,B). The NK–DC cross talk greatly increased NK cell killing compared to the single cultured NK cells, independent on the treatment (Figures 5A,B). The HIV-exposed DCs activated less cytotoxicity in the NK cells compared to mock (Figures 5A,B). C-HIV and CI-HIV exposure led to a significantly lower killing ability than that F-HIV exposure. The amount of target cells killed by NK cells from cross talk cultures exposed to complement-opsonized virus decreased by approximately 50% (Figures 5A,B). The DCs were protected from the NK cell cytotoxicity as the killing of these cells was quite low (below 2%), with a very low but significant decrease for DCs exposed to C-HIV (Figure 5C).

### DCs Exposed to HIV and Conditioned by NK Cells Suppressed T Cell Proliferation and Promoted the Differentiation of Central Memory T Cells

**Figure 6:**
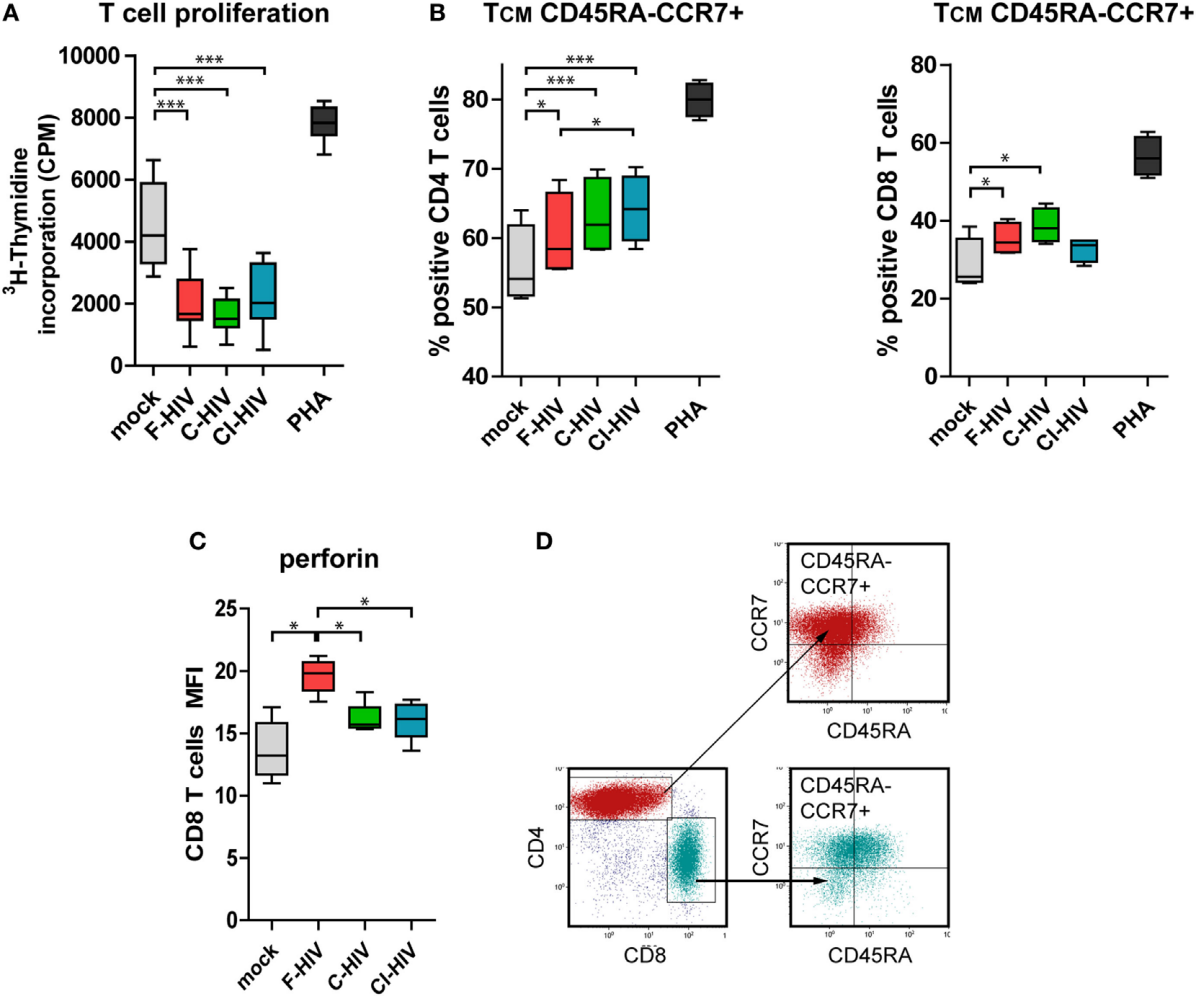
T cell proliferation and memory phenotype activated by natural killer (NK) cell–dendritic cell (DC) stimulation assay. DCs and NK cells from the same donor were exposed to 1 μg/ml F-HIV, complement-opsonized HIV (C-HIV), complement- and antibody opsonized HIV (CI-HIV), PHA, or mock-treated for 24 h. The experiment was replicated four times using cells derived from four different donors. Allogeneic T cells were stimulated by cells from these cultures at a 1:10 DC:T cell ratio. **(A)** T cell proliferation was assessed using a ^3^H-Thymidine incorporation assay. **(B)** Supernatants from the NK–DC cross talk cultures were added to allogeneic T cells stimulated by CD3/CD28 ligation and the percentage of cells with central memory (T_CM_) phenotype in the CD4 and CD8 T cell populations was determined using flow cytometry. **(C)** The amount of perforin in the CD8 T cells from the NK–DC stimulation assay was evaluated. **(D)** Gating strategy for flow cytometry analysis of a representative sample can be seen in. Boxplots show mean with Tukey error bars. One-way ANOVA followed by a Bonferroni posttest was used to test for statistical significance (*p < 0.05, *N* = 4).

Next, we assessed the quality and type of T cell responses activated by DCs conditioned by NK (NK–DC cross talk) in the presence of mock, F-HIV, C-HIV, or CI-HIV. Naïve allogeneic T cells were isolated and added to the NK–DC cross talk cultures. T cell proliferation was assessed by ^3^H-thymidine incorporation assay. DCs exposed to both the free and C-HIV significantly suppressed the proliferation of T cells (Figure 6A). The profiles were similar for T cells exposed to DCs cultured both in single culture and DCs conditioned by NK cells (Figure 6A and data not shown). In the naïve T cells primed by CD3/CD28 beads in the presence of supernatant from NK–DC cross talk cultures, the CD4 and CD8 subsets differentiated into central memory T cells (T_CM_, CD45RA-CCR7+), with higher levels of T_CM_ T cells in the virus exposed groups, and with C-HIV and CI-HIV leading to a higher increase than F-HIV (Figures 6B,C; Figures S1A,B in Supplementary Material). Gating strategy for the T_CM_ flow cytometry analysis can be seen in Figure 6D. In order to assess whether the T cells primed by DCs conditioned by NK cells had acquired effector functions, the T cell expression of perforin was evaluated by flow cytometry and all HIV conditions induced perforin and granzyme B expression in approximately 80% of the CD8 T cells (Figure S2 in Supplementary Material). The amount of perforin per cell was higher in CD8 T cells activated by NK-conditioned DCs exposed to F-HIV compared to C-HIV, CI-HIV, or mock (Figure 6B), indicating that C-HIV and CI-HIV could suppress the cytolytic responses in T cells as well as in NK cells.

### Cells From NK–DC Cross Talk Cultures Exposed to C-HIV Induced CXCR3+ CCR4+ CD4 T Cells

**Figure 7:**
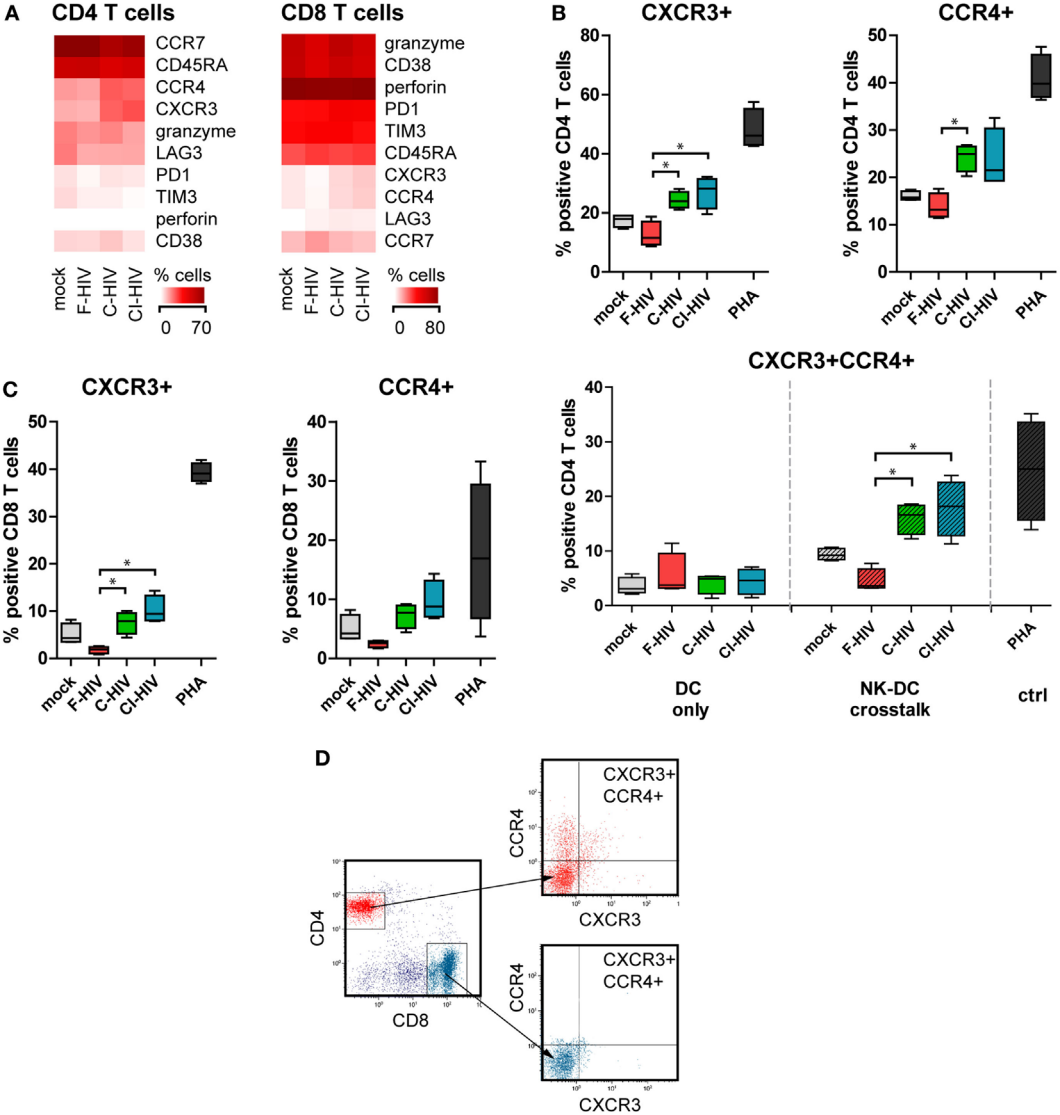
T cell phenotype activated by natural killer (NK)–dendritic cell (DC) stimulation assay. DCs and NK cells from the same donor were cocultured and exposed to 1 μg/ml F-HIV, complement-opsonized HIV (C-HIV), complement- and antibody opsonized HIV (CI-HIV), PHA, or mock treated for 24 h. The experiment was replicated four times using cells derived from four different donors. Allogeneic T cells were then added to the cultures at a 1:10 DC:T cell ratio. **(A)** The T cell phenotype induced was assessed by flow cytometry. Heat maps of the percentage of CD4 and CD8 cells positive for phenotypic markers were created. **(B)** The number of CD4 T cells positive for CXCR3 or CCR4 were evaluated in culture with DCs conditioned with NK cells, and the number of CD4 T cells positive for both CXCR3 and CCR4 were evaluated in culture with DCs conditioned with NK cells or DCs alone. **(C)** The number of CD8 T cells positive for CXCR3 and CCR4 was evaluated in culture with DCs conditioned with NK cells. **(D)** Gating strategy for flow cytometry analysis of a representative sample can be seen. Boxplots show mean with Tukey error bars. One-way ANOVA followed by a Bonferroni posttest was used to test for statistical significance (*p < 0.05, *N* = 4).

Naïve allogeneic T cells that were added to NK–DC cultures were stained for an array of phenotypic markers and assessed by flow cytometry (Figure 7A). Expression levels of CXCR3 and CCR4 in CD4 T cells were significantly upregulated when the cross talk cultures had been exposed to C-HIV or CI-HIV but not to F-HIV (Figures 7A,B). In addition, C-HIV and CI-HIV significantly increased the level of CXCR3+ CCR4+ CD4 T cells, whereas F-HIV decreased the level compared to mock (Figure 7B). The upregulation of CXCR3 and CCR4 by NK–DC cross talk cultures exposed to C-HIV or CI-HIV was also seen for CD8 T cells, although receptor expression was induced in a lower number of cells (Figure 7C). The upregulation of CXCR3 and CCR4 expression on T cells by C-HIV was only achieved in the by NK–DC cross talk cultures, but not when the T cells were merely activated by DCs exposed to HIV (Figure 7B). This indicates that the effect is likely dependent on contact-mediated interaction with NK cells or both DCs and NK cells. The flow cytometry gating was first performed on singlet viable CD3+ T cells followed by gating on CD4 or CD8 positive cells and finally by assessing the expression levels of CCR4 and CXCR3 on these cells. The gating strategy for flow cytometry analysis is presented in Figure 7D.

### DCs Exposed to C-HIV Upregulated Negative Costimulatory Molecule Expression on CD4 T Cells

**Figure 8:**
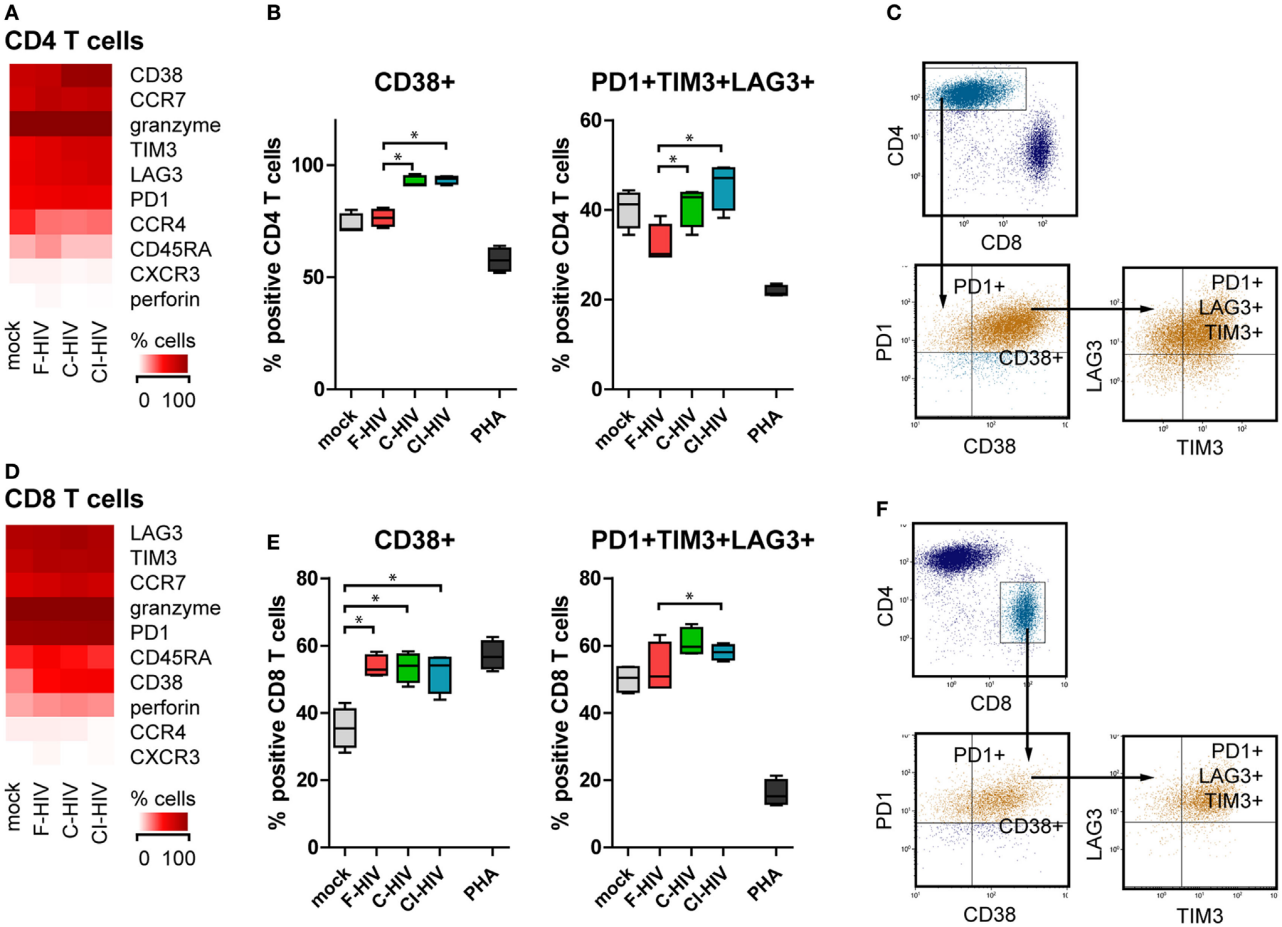
Expression of immune checkpoint factors on the CD4 and CD8 T cells activated by CD3 and CD28 ligation and supernatants from the natural killer (NK)–dendritic cell (DC) cross talk assay. DCs and NK cells from the same donor were exposed to 1 μg/ml F-HIV, complement-opsonized HIV (C-HIV), CI-HIV, PHA, or mock treated for 24 h. The experiment was replicated four times using cells derived from four different donors. Allogeneic T cells were stimulated by CD3/CD28 ligation in the presence of supernatants from these cultures. **(A,D)** The CD4 T cell **(A)** and CD8 T cell **(D)** phenotype generated was assessed using flow cytometry. Heat maps of the percentage of CD4 cells or CD8 T cells positive for phenotypic markers was created. The number of CD4 T cells **(B)** or CD8 T cells **(E)** positive for CD38, and the number of cells coexpressing PD-1, TIM-3, and LAG-3 were evaluated. **(C,F)** Gating strategy for flow cytometry analysis of a representative sample. Boxplots show mean with Tukey error bars. One-way ANOVA followed by a Bonferroni posttest was used to test for statistical significance (*p < 0.05, *N* = 4).

Supernatants were harvested from to NK–DC cultures exposed to different forms of HIV or mock treated and added to allogenic T cells activated by CD3 and CD28 ligation. The T cell phenotype was assessed by flow cytometry. CD3 and CD28 ligation together with supernatants from cultures exposed to C-HIV and CI-HIV induced CD4 T cell upregulation of the activation marker CD38 (Figures 8A,B), whereas supernatants from cultures exposed to F-HIV did not. All virus conditions led to the upregulation of the co-inhibitory molecules PD-1, TIM-3, and LAG-3 on CD4 T cells, with the highest upregulation seen for C-HIV and CI-HIV (Figure 8A; Figure S3 in Supplementary Material). The populations of CD4 T cells that were positive for PD-1 or PD-1 in combination with TIM-3 and LAG-3 were significantly higher when activated with CD3 and CD28 ligation and exposed to supernatants from C-HIV and CI-HIV than from F-HIV (Figure 8B; Figure S3 in Supplementary Material). The expression of the inhibitory molecules on CD8 T cells was similar to that for CD4 T cells described above, but less pronounced. In addition, the T cells activated by CD3 and CD28 ligation and exposed to C-HIV or CI-HIV supernatants induced a larger population of CD8 T cells that coexpressed PD-1, TIM-3, and LAG-3 (Figures 8D,E). In addition, the CD3 and CD28 ligation activated CD8 T cells upregulated CD38 upon all virus exposures, whereas CD4 T cells only upregulated CD38 in the presence of supernatants derived from cultures exposed to C-HIV and CI-HIV (Figures 8B,E). Noteworthy, PHA stimulation led a substantial increase in amount of PD-1 positive cells (Figures S2 and S3 in Supplementary Material), whereas the percentage of cells coexpressing PD-1, TIM-3, and LAG-3 was decreased. PHA did not induce a high upregulation of CD38; which could be due to the expression kinetics of this marker (38, 39). The gating strategy for CD38, PD-1, TIM-3, and LAG-3 on CD4 and CD8 T cells is presented in Figures 8C,F.

## Discussion

Dendritic cells, NK cells, and T cells at the mucosal linings all have important roles during HIV exposure and interactions between these critical immune cells are likely to determine whether the exposure leads to establishment of HIV infection as well as shaping the immune responses against the virus. In this study, we investigated how free HIV (F-HIV), and HIV opsonized with complement only (C-HIV), or with both complement and antibodies (CI-HIV) affected DCs and NK cells alone and during cross talk, and how this cellular cross talk in turn affected their ability to activate naïve T cells and the T cell phenotypes generated.

Our transcriptome profiling and flow cytometry experiments revealed that C-HIV induced lower activation of DC maturation pathways and factors compared to free HIV and also less NK cell activation during NK–DC cross talk. We have previously shown that C-HIV inhibits inflammatory and antiviral responses in DCs in a CR3-dependent manner (12), and in this study, the suppression of factors associated with regulating responses to danger was clearly visible both for DCs cultured alone and for DCs derived from NK–DC cross talk experiments. Danger signaling is tightly linked to DC maturation (40), as well as their ability to activate NK cells (41). Even if the NK cells in single culture were activated by HIV, it was apparent that the responses induced by HIV in the DCs defined the NK responses since the transcriptomes of the DCs were considerably less affected by the cross talk than the transcriptomes of the NK cells. In addition, the presence of C-HIV did not suppress NK cell activation in the absence of DCs. These findings support the concept of the DC as the determining factor during HIV transmission (5). Of note, *in vivo* studies have also described that during acute infection, DCs produce reduced amounts of IL-12, IL-15, and IL-18, leading to lower IFN-γ production by NK cells, which consequently results in poor DC maturation (21, 42).

Natural killer cells have the ability to recognize and kill virus infected target cells by the release of perforin and granzymes, or engagement of target cell death receptors such as Fas by Fas-L (43). NK-mediated killing creates a substantial immune pressure on HIV (44), and it has been suggested that the ability of NK cells to eliminate infected cells from the primary site of HIV infection can avert systemic infection (2, 42). In this study, DCs exposed to HIV suppressed the pathways involved in the activation of the NK cell ability to kill target cells, which is in accordance with *in vitro* studies by others (25). The effect DCs exposed to C-HIV had on the NK cell killing was striking as it severely suppressed this important NK effector function, with less than half the number of target cells killed. This dexterous effect by C-HIV on the NK cell’s cytotoxic function should impair the ability of the host to control the HIV infection.

Dendritic cell and NK cell communications occur in the presence of HIV both locally in tissues as well as in the lymphoid system (45, 46). The ability of NK cells to lyse immature DCs in order to avoid defective T cell priming is known as “DC-editing,” and this has been shown to enhance the expansion of antigen specific cytotoxic T cells (47), which are important effector cells involved in controlling the HIV infection. However, the capacity of NK cells to carry out DC-editing is reduced by HIV *in vivo* (21, 48). NK cell lysis of immature DCs has been shown to involve the NKp30 and DNAM-1 receptors whereas the upregulation of MHC class I on activated DCs can protect them from NK killing (26). In our system, DCs exposed to C-HIV or CI-HIV inhibited NK killing of both target cells and of the DCs themselves. Of note, RNAseq analysis revealed that several upstream regulators that promote survival were upregulated in the DCs, i.e., the survival of DCs could be due to both suppressed NK cytotoxicity and a consequence of higher resistance to killing in the DCs. The precise mechanisms involved in the suppression of NK killing by DCs exposed to C-HIV remain to be elucidated. Interestingly, in our system the upregulation of perforin levels in CD8 T cells exposed to NK–DC cultures was also inhibited by C-HIV and CI-HIV, i.e., it is possible that the DC-mediated inhibition of cytotoxic responses when exposed to C-HIV occurred through pathways common to both NK cells and CD8 T cells.

The effects HIV exerted on the NK-conditioned DCs’ ability to prime and activate naïve T cells were assessed in HIV-exposed NK–DC cell cross talk cultures. All three forms of HIV directed naïve T cells to differentiate to central memory (CCR7+ CD45RA−) T cells. The DCs exposed to C-HIV or CI-HIV induced a slightly higher amount of CD4 and CD8 T cells with central memory phenotype, which indicates that the presence of complement did not interfere with the T cell differentiation from naïve to memory T cells, rather increase the pool of central memory T cells. Of note, central memory CD4 T cells are permissive to HIV infection and can form latent reservoirs (49) with replication-competent virus (50), and their induction and quantity is, therefore, highly relevant to HIV pathogenesis. Noteworthy, exposure to complement-opsonized virus was associated with lower levels of effector memory T cells than exposure to free virus, which could explain the higher perforin expression seen for F-HIV as perforin expression is known to be higher in effector memory T cells (51).

The effect of DC exposure to HIV on the T cell proliferation was massive with the proliferation of T cells stimulated by HIV-exposed cultures reduced to approximately 50% compared to T cells exposed to mock treated cultures. There was no significant difference in viral T cell suppression between the free and complement-opsonized virus. The reduction in DC’s capacity to induce T cell proliferation after HIV exposure has been described previously, by our group and by others (52, 53), but the effect of complement has not been investigated previously.

Negative immune checkpoint molecules, such as PD-1, LAG-3, TIM-3, and PD-L1, were expressed to a higher extent on the T cells and DCs cultured in the presence of C-HIV than on cells exposed to free virus. In addition, stimulation of T cells by supernatants from NK–DC cross talk cultures exposed to C-HIV or CI-HIV led to a higher amount of both CD4 and CD8 T cells coexpressing PD-1, LAG-3, and TIM-3. The expression of PD-1 is hallmark of an exhausted T-cell phenotype with limited ability to respond to stimuli (54, 55). Exhausted T cells are characterized by a loss of proliferative capacity and cytotoxic activity (56–58). At an initial stage, the PD-1 pathway only dampens the CD8 T cell responsiveness, but can eventually lead to a hierarchical loss of proliferation, cytolytic activity, defects in cytokine production, and eventually deletion (57). A synergistic effect between PD-1 and other negative checkpoint factors has been described (59), and T cells coexpressing PD-1, TIM-3, and LAG-3 are often even more exhausted than cells expressing only PD-1 (57). While PD-1 blockade can reverse exhaustion in cells expressing PD-1 only, this is not true when TIM-3 and LAG-3 are coexpressed on the PD-1 positive cells, which indicates the existence of multiple redundant T cell suppression pathways (57). Consequently, the higher expression of both PD-1 itself, as well as the coexpression of TIM-3 and LAG-3, on T cells stimulated by cultures exposed to complement-opsonized virus is likely to have detrimental effects on immune function, thereby contributing to HIV pathogenesis.

Another factor that is an indicator of disease progression and dysfunctional T cells in untreated HIV infection is CD38. Expression of this activation marker is high on activated T cells, low on naïve T cells, and undetectable on resting memory T cells (60). Low number of CD4 and CD8 T cells expressing CD38 was associated with reduced permissiveness to HIV replication in cervical explants (61), indicating that CD38 expression either supports or is a product of HIV infection in the mucosa. We found CD38 expression to be upregulated on CD8 T cells primed and activated by CD3 and CD28 ligation following exposure to supernatants from all HIV-exposed NK–DC cross talk cultures, whereas CD4 T cells upregulated CD38 upon exposure to supernatant from C-HIV or CI-HIV cultures only. Recent observations suggest that CD38 plays an active role in HIV infection and in chronic HIV infection *in vivo*, where increased expression of CD38 on CD8 T cells appears to be associated with immune activation and HIV disease progression (60). In addition, HLADR+ CD38+ CD4 T cells have been shown to produce high levels of HIV due to higher levels CCR5 and CXCR4 (39), indicating that CD4 T cells primed by DCs exposed to C-HIV could support a higher HIV replication.

The ability to migrate to the site of infection and inflammation or into the lymph is highly relevant for most immune cells and proper migration involves an array of chemokine receptors. CD4 T cells activated by NK–DC cross talk cultures exposed to C-HIV had higher expression levels of CCR4 and CXCR3, than CD4 T cells stimulated by NK–DC exposed to free HIV. The same was also true for the CD8 T cells, although the number of CCR4 and CXCR3 positive cells was much lower than in the CD4 population. CCR4 and CXCR3 positive T cells have been shown to traffic to and infiltrate inflamed tissue (62, 63). CXCR3 expression is considered to be a signature of gut-homing potential in CD4 T cells (63). In lung, CTL migration to infection sites has been shown to be CXCR3 dependent (64), and this tissue positioning is considered to be one of the rate-limiting steps in CTL-mediated protection (65). CXCR3+ and CCR4+ CD4 T cells are highly permissive to HIV infection and replication (66). In chronic SIV infection, there are increased levels of CXCR3 positive CD4 T cells but not of CCR4 positive CD4 T cells and this is also true in the lymph nodes, where the CXCR3 positive T follicular helper cells levels are associated with high viral loads (67).

CXCR3 expression has been shown to be induced by IFN-γ inducible ligands CXCL9, CXCL10, and CXCL11 (62). In our system, CXCL9 could play a role in the NK mediated induction of CXCR3 expression, as concentrations of this cytokine were highest in the NK–DC cross talk cultures exposed to C-HIV and CI-HIV. Both DCs and NK cells have the ability to produce CXCL9 (68). The CXCL9 mRNA expression levels was much higher in the DCs compared to the NK cells from the cross talk cultures, indicating that it is likely that the DCs account for the majority of CXCL9 production in our system. The levels of CXCL10 were upregulated in the NK–DC cross talk system by all HIV conditions. Interestingly, the upregulation of CXCR3 and CCR4 by C-HIV only occurred when both DC and NK cells were present during the T cell stimulation. Direct CD4 T cell–NK cell interactions stimulate upregulation CXCR3 on the CD4 T cells by means of IL-21 (69). However, our RNAseq did not detect any IL-21 mRNA expression, indicating that there are other mechanisms at that require direct interactions with both the DCs and NK cells during T cell conditioning, which remain to be investigated.

**Figure 9:**
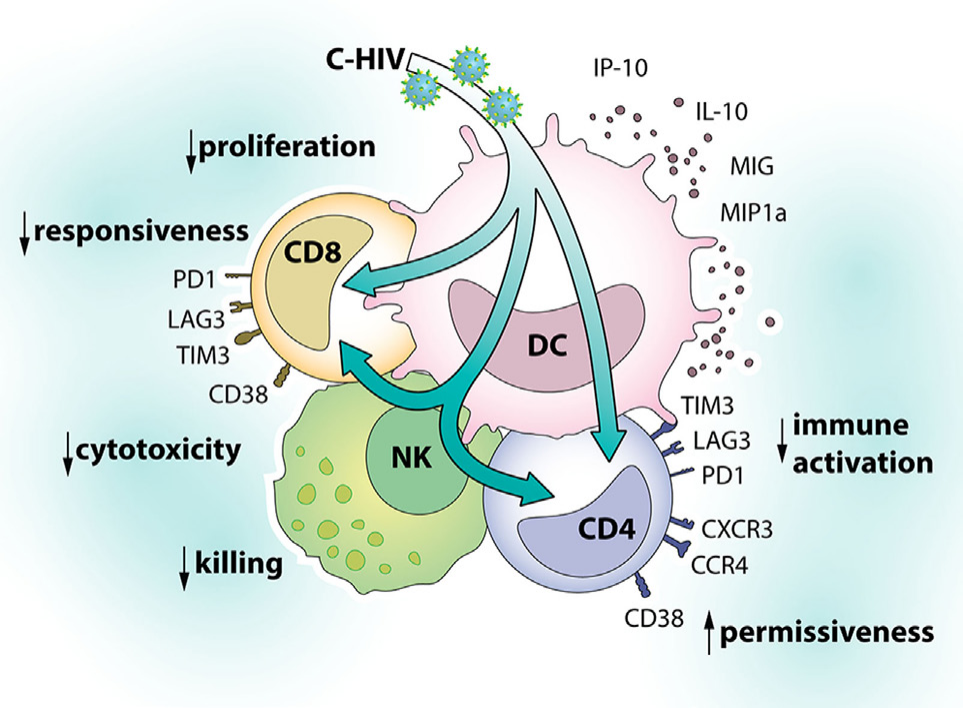
Effects of complement-opsonized HIV (C-HIV) on dendritic cells (DCs), natural killer (NK) cells, and T cell phenotype. Complement fragments on HIV’s surface modulate responses in DCs and their cross talk with NK cells to inhibit killing and promote the upregulation of factors associated with immune suppression (PD-1, TIM-3, LAG-3) and susceptibility to infection (CD38, CXCR3, CCR4) on T cells.

CCR4+ CCR5+ CD4 T cells are highly permissive to HIV infection and CCR4 is expressed by T helper type 2 and type 17 cells and also by regulatory CD4 T cells (66). Similar to CXCR3, CCR4 is important for the migration of T cells to sites of inflammation or infection (70). In addition, it has been suggested that CCR4 promotes retention of T cells in different tissues (71). HIV is highly dependent on availability of permissive cells for transmission and spread (28). The induction of a CD4 phenotype that is permissive to infection and has gut-homing capabilities, therefore, likely contribute to HIV infection and spread. An overview of the effects of C-HIV on DCs, NK cells, and T cell phenotype can be found in Figure 9.

In summary, presence of complement fragments on HIV’s surface modulated responses in DCs and their cross talk with NK cells to inhibit killing and to promote the upregulation of factors associated with immune suppression (PD-1, TIM-3, LAG-3) and susceptibility to infection (T_CM_, CD38, CXCR3, CCR4) on CD4 T cells. Complement opsonization, therefore, likely contributes to HIV transmission and pathogenesis.

## Author Contributions

Conception/design: RE and ML. Data collection: RE, MK, HH, YT, and MW. Data analysis/interpretation: RE and MK. Drafting article: RE, ML, and ES. Critical revision of the article: CS, JH, SN, and ES. Final approval of the version to be published: RE, MK, CS, HH, YT, MW, JH, SN, ES, and ML.

## Conflict of Interest Statement

The authors declare that the research was conducted in the absence of any commercial or financial relationships that could be construed as a potential conflict of interest.
