# Supplementary material for: Complement-Opsonized HIV-1 Alters Cross Talk Between Dendritic Cells and Natural Killer (NK) Cells to Inhibit NK Killing and to Upregulate PD-1, CXCR3, and CCR4 on T Cells
Source: Front Immunol. 2018 Apr 30;9:899. doi: 10.3389/fimmu.2018.00899 (PMC5936988; doi:10.3389/fimmu.2018.00899)
Supplement: Supplementary file 1 [file data_sheet_1.PDF]

Supplementary Material

Complement Opsonized HIV-1 Alters Crosstalk between Dendritic Cells and NK Cells to Inhibit NK Killing and to Upregulate PD-1, CXCR3 and CCR4 on T Cells

Rada Ellegård[1], Mohammad Khalid[1], Cecilia Svanberg[1], Hanna Holgersson[1], Ylva Thorén[1], Mirja Wittgren[1], Jorma Hinkula[1], Sofia Nyström[1,2], Esaki M. Shankar[3,4,5] and Marie Larsson[1*]

*** Correspondence:** Professor Marie Larsson: marie.larsson@liu.se

Supplementary Table 1. Top upstream regulators in DCs. DCs (N=3) were exposed to 1µg/ml F-HIV, C-HIV, CI-HIV or mock treated for 3h. NK cells from the same donor were then added at a 1:1 ratio and the crosstalk cultures were incubated for an additional 21h. DCs from the cultures were purified and RNAseq was performed. The RNAseq data was normalized to the mock treated sample using DeSeq2/R then analyzed using IPA (Qiagen) to determine which upstream activators were upregulated in the different conditions. The top regulators were then divided into functional groups manually.

Upstream regulator	F-HIV	C-HIV	CI-HIV	group
TNF	2.53	-3.83	-3.69	danger
RABL6	-2.65	3.77	3.21	growth
NUPR1	0.35	-4.95	-3.92	growth
TP53	-1.23	4.77	3.57	growth
ERBB2	-2.50	0.72	0.57	growth
IFNG	1.99	-1.25	-0.12	danger
TAL1	0.38	4.17	3.79	growth
MITF	-0.86	4.03	3.70	growth
IL1B	1.57	-2.37	-0.94	danger
Jnk	-0.43	-1.21	-1.06	danger
NFkB (complex)	1.65	-3.72	-2.30	danger
CDKN1A	1.02	-2.77	-2.77	growth
PDGF BB	-1.13	-1.33	-0.16	growth
HSF1	2.24	0.24	-0.20	danger
IL1A	1.75	-2.29	-0.90	danger
AREG	-2.12	2.04	2.74	growth
TGFB1	1.14	-0.98	-0.85	growth
ERK1/2	-0.57	-0.99	-1.34	growth
TGM2	0.27	1.90	2.72	growth
IL17A	1.26	-0.48	0.47	danger
PRKCD	-1.15	2.76	3.10	growth
Akt	1.33	0.88	-0.41	danger
SYK	-0.47	2.62	3.08	danger
TREM1	-1.21	-0.72	-1.13	danger
STAT1	1.31	-3.05	-2.24	danger
BRCA1	0.00	1.94	2.40	growth
TLR7	0.81	-1.36	-0.25	danger
GLI1	0.05	-1.99	-2.19	growth
IL33	0.00	-2.23	-1.53	danger
SMARCA4	1.60	-1.72	-0.96	transcription
RELA	1.16	-1.85	-0.19	danger
FOXM1	-0.97	1.90	2.47	transcription
PGR	-0.23	-2.43	-2.11	transcription
SOX11	-1.08	-0.40	0.42	transcription
SIRT1	-0.45	1.27	2.19	transcription
UXT	0.21	-3.80	-1.72	danger
E2F3	-1.21	1.80	1.20	growth
IL2	-0.09	-2.29	-1.49	danger
E2F6	0.00	2.45	2.00	growth
AR	-2.48	-3.84	-2.76	transcription
KIAA1524	-1.67	2.12	2.12	growth
BRD4	-0.82	-1.52	-1.52	transcription
ECSIT	1.60	-1.66	-1.66	danger
MAPK1	-1.09	-0.22	-1.29	growth
PPARA	0.00	1.95	1.95	transcription
MGEA5	0.00	1.04	0.94	transcription
CBX5	0.71	2.54	1.93	transcription
Mek	-0.55	0.25	0.38	growth
CD40LG	0.89	-2.46	-1.99	danger
KLF6	0.00	-0.62	-0.65	growth
PPP2R5C	-0.65	2.00	2.00	growth
CDKN2A	0.00	2.79	1.47	growth
IL13	2.65	-0.83	-0.57	danger
IGF1	1.20	-0.81	-0.55	growth
ATM	0.19	-0.64	-1.24	growth

Supplementary Table 2. Top upstream regulators in NK cells. DCs (N=3) were exposed to 1μg/ml F-HIV, C-HIV, CI-HIV or mock treated for 3h. NK cells from the same donor were then added at a 1:1 ratio and the crosstalk cultures were incubated for an additional 21h. NK cells from the cultures were purified and RNAseq was performed. The RNAseq data was normalized to the mock treated sample using DeSeq2/R then analyzed using IPA (Qiagen) to determine which upstream activators were upregulated in the different conditions. The top regulators were then divided into functional groups manually.

Upstream regulator	F-HIV	C-HIV	CI-HIV	group
PRL	0.14	0.05	0.33	growth
IFNG	0.66	-0.83	-0.03	IFN
IFNA2	-0.12	-0.38	-0.39	IFN
IFNL1	-0.04	-0.24	-0.08	IFN
IL1RN	0.00	0.42	0.26	inflammation
TGM2	0.35	0.98	0.13	growth
TNF	0.16	-0.05	-0.11	inflammation
CSF2	0.73	0.20	0.00	growth
TLR7	-0.01	-0.29	0.28	IFN
STAT1	0.54	-0.38	-0.57	IFN
EIF2AK2	0.25	-0.70	0.25	IFN
SYVN1	0.63	0.81	0.64	growth
TLR9	0.03	-0.48	-0.12	IFN
P38 MAPK	-0.14	0.07	-0.36	growth
CCL5	0.24	0.12	0.46	inflammation
PAF1	0.00	-0.24	-0.47	growth
MAPK1	-0.04	0.91	0.40	growth
IL13	0.34	0.70	0.50	inflammation
NFkB (complex)	-0.73	-0.67	-0.24	inflammation
TLR4	-0.26	-0.43	0.01	inflammation
IL5	0.53	0.21	-0.24	inflammation
EGFR	0.03	0.17	0.02	growth
ERG	0.56	0.29	0.15	growth
IFNB1	-0.17	-0.25	0.00	IFN
Mek	-0.30	0.41	0.07	growth
CD40LG	0.00	-0.22	0.02	inflammation
GLI1	0.12	1.35	0.95	growth
BRD4	-0.24	0.40	-0.24	growth
TGFB1	0.69	0.07	1.02	inflammation
Interferon alpha	-0.70	-2.08	-1.11	IFN
STAT3	0.41	0.74	0.57	growth
IL4	-0.50	-0.35	-0.20	inflammation
TAB1	0.16	0.17	0.00	growth
CSF1	-0.01	0.16	0.96	growth
ERK	-0.40	0.63	0.65	growth
TLR2	-0.88	-0.68	-0.22	inflammation
EHF	-0.18	-0.50	-0.50	growth
ERK1/2	-0.40	-0.30	0.42	growth
SELPLG	-0.40	0.60	0.00	inflammation
MAPK14	-0.56	0.00	-0.16	growth
SOCS1	-0.17	0.19	0.00	inflammation
KDM5B	-0.23	-0.13	-0.76	growth
IL6	0.42	0.01	-0.59	inflammation
IFNAR2	0.00	-0.85	-0.36	IFN

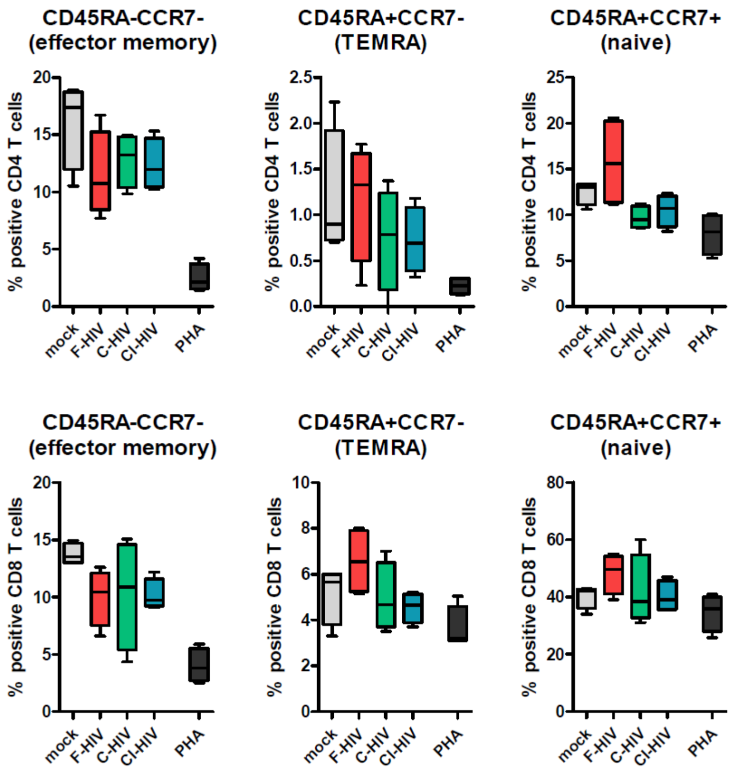

Supplementary Figure 1. Phenotype of T cells stimulated by CD3/CD28. DCs (N=4) were exposed to 1µg/ml F-HIV, C-HIV, CI-HIV, PHA or mock treated for 3h. The DCs were then either kept as a single culture or in a crosstalk culture with NK cells from the same donor at a 1:1 ratio for an additional 21h. The supernatants were harvested and added to allogeneic T cells stimulated by CD3 and CD28 ligation for 24h. Percentage of CD4 (A) or CD8 (B) T cells positive for phenotypic markers CD45RA and CCR7 was determined using flow cytometry.

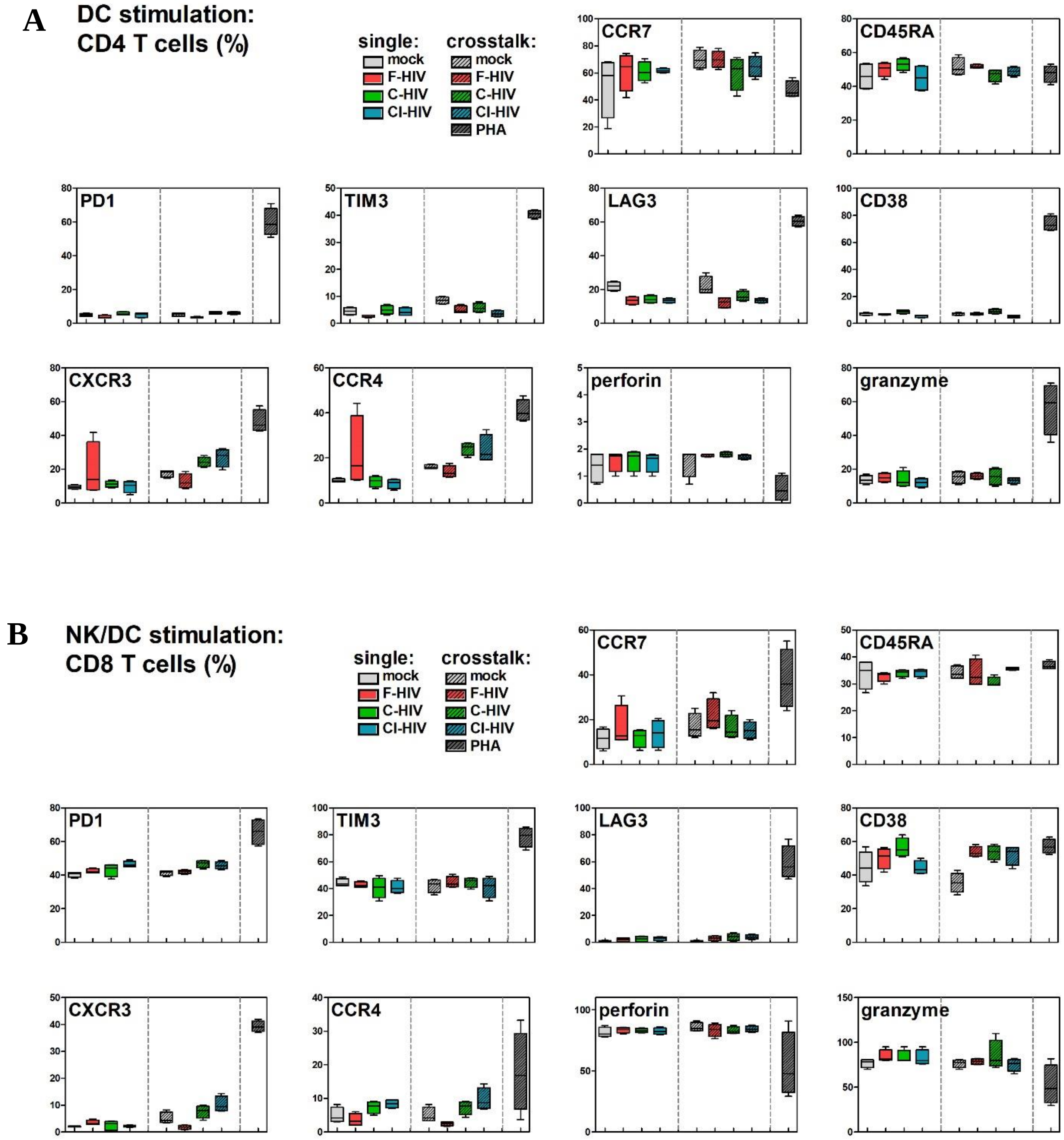

Supplementary Figure 2. Phenotype of T cells stimulated by NK/DC. DCs (N=4) were exposed to 1µg/ml F-HIV, C-HIV, CI-HIV, PHA or mock treated for 3h. The DCs were then either kept as a single culture or in a crosstalk culture with NK cells from the same donor at a 1:1 ratio for an additional 21h. The DCs and NK cells were harvested from the cultures and used to stimulate allogeneic naïve T cells at a 1:10 ratio for 24h. Percentage of CD4 **(A)** or CD8 **(B)** T cells positive for phenotypic markers was determined using flow cytometry.

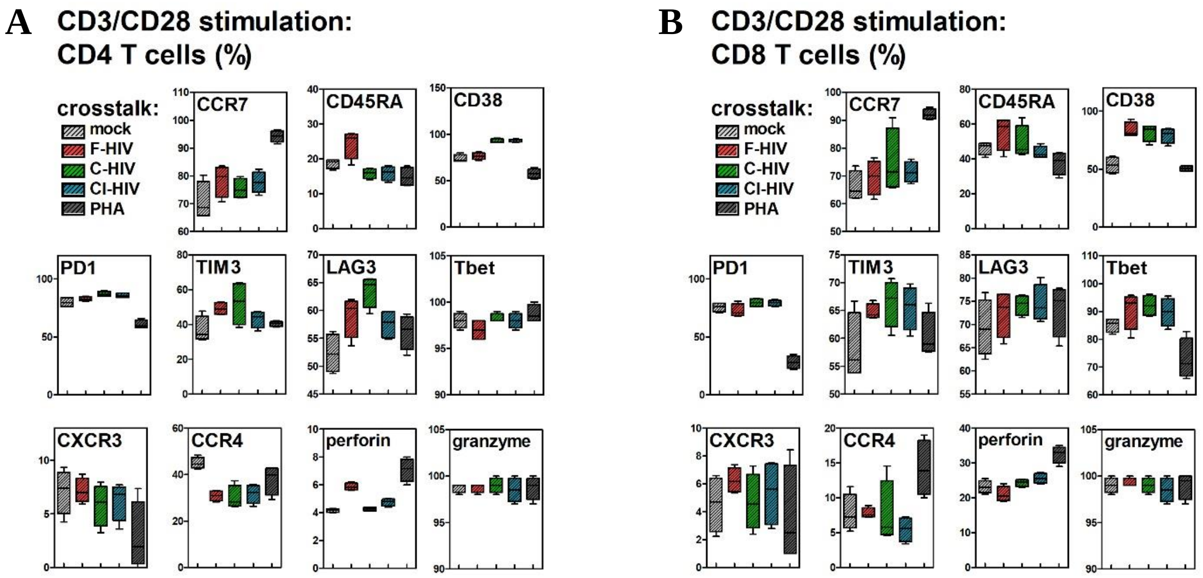

Supplementary Figure 3. Phenotype of T cells stimulated by CD3/CD28. DCs (N=4) were exposed to 1μg/ml F-HIV, C-HIV, CI-HIV, PHA or mock treated for 3h. The DCs were then either kept as a single culture or in a crosstalk culture with NK cells from the same donor at a 1:1 ratio for an additional 21h. The supernatants were harvested and added to allogeneic T cells stimulated by CD3 and CD28 ligation for 24h. Percentage of CD4 **(A)** or CD8 **(B)** T cells positive for phenotypic markers was determined using flow cytometry.
